# Effects of Neonicotinoid Insecticides on Physiology and Reproductive Characteristics of Captive Female and Fawn White-tailed Deer

**DOI:** 10.1038/s41598-019-40994-9

**Published:** 2019-03-14

**Authors:** Elise Hughes Berheim, Jonathan A. Jenks, Jonathan G. Lundgren, Eric S. Michel, Daniel Grove, William F. Jensen

**Affiliations:** 1 Department of Natural Resource Management, South Dakota State University, Brookings, SD USA; 2 Ecdysis Foundation, Estelline, SD 57234 USA; 3 North Dakota Game and Fish Department, Bismarck, ND USA

## Abstract

Over the past decade, abnormalities have been documented in white-tailed deer (*Odocoileus virginianus*) in west-central Montana. Hypotheses proposed to explain these anomalies included contact with endocrine disrupting pesticides, such as imidacloprid. We evaluated the effects of imidacloprid experimentally at the South Dakota State University Wildlife and Fisheries Captive Facility where adult white-tailed deer females and their fawns were administered aqueous imidacloprid (an untreated control, 1,500 ng/L, 3,000 ng/L, and 15,000 ng/L). Water consumption, thyroid hormone function, behavioral responses, and skull and jawbone measurements were compared among treatments. Additionally, liver, spleen, genital, and brain imidacloprid concentrations were determined by an enzyme-linked immunosorbent assay (ELISA). Results indicated that 1) control deer consumed more water than treatment groups, 2) imidacloprid was present in the organs of our control group, indicating environmental contamination, 3) as imidacloprid increased in the spleen, fawn survival, thyroxine levels, jawbone lengths, body weight, and organ weights decreased, 4) adult female imidacloprid levels in the genitals were negatively correlated with genital organ weight and, 5) behavioral observations indicated that imidacloprid levels in spleens were negatively correlated with activity levels in adult females and fawns. Results demonstrate that imidacloprid has direct effects on white-tailed deer when administered at field–relevant doses.

## Introduction

Neonicotinoids are a broad-spectrum insecticide predominantly used as seed dressings on major field crops and are additionally used as sprays in crop production, in managing household pests, and in deterring pests on domesticated animals^1^. Neonicotinoids derive their toxicity from agonistically binding to nicotinic acetylcholine receptors (nAChRs) on the post-synaptic nerve membrane and firing nerve impulses in a manner that is uncontrollable and uninterrupted^1–7^. Neonicotinoids were first developed in the 1990s^8^, gained popularity from 2003-2011^9^, and are now the most widely used pesticides in the world^10^.

Popularity of neonicotinoids is due to their advertised high toxicity to insects and low toxicity to vertebrates^1^. Additionally, neonicotinoids have gained popularity by their ability to systemically protect plants while reducing application inputs for farmers^10^. In 2014, over 3.3 million kg of neonicotinoids (including acetamiprid, clothianidin, dinotefuran, imidacloprid, thiacloprid, and thiamethoxam) were used in the United States (excluding Hawaii and Alaska) on pasture hay, alfalfa, orchards, grapes, rice, vegetables, fruit, cotton, wheat, soybeans, corn, and other crops^11^. In South Dakota, more than 94% of U.S. corn and at least 50% of U.S. soybeans^12^ are treated with one of the three neonicotinoids: clothianidin, imidacloprid, or thiamethoxam^13–15^.

Neonicotinoids are widely found in the environment for numerous reasons. First, only a small quantity (2–20%) of the seed-coated insecticide is absorbed by the developing plant; the remainder is released into the environment through leaching, drainage, run-off, or snowmelt^16,17^. Neonicotinoids are highly water soluble^18^; they are prevalent in diverse water bodies in the United States, Canada, Australia, Europe, and Asia^17^. Moreover, under the right conditions, neonicotinoids can persist in the soil, sometimes for many years^1^. Finally, untreated plants associated with cropland are often contaminated by neonicotinoids due to the systemic nature of these chemicals^19^. The widespread use of neonicotinoids provides numerous opportunities for exposure to non-target, beneficial species via the water, soil, and contaminated plant tissues.

In addition to their documented effects on beneficial insects, neonicotinoids adversely affect non-target vertebrates as well, including rats (*Rattus norvegicus*: reduced sperm production, reduced offspring weight, increased abortions, skeletal abnormalities, thyroid lesions, atrophy of retina, reduced weight gain of offspring, oxidative stress, and neurobehavioral deficits), mice (*Mus musculus*: suppressed cell-mediated immune response and prominent histopathological alterations in spleen and liver), rabbits (*Sylvilagus* sp: increased frequency of miscarriage and premature births), red-legged partridges (*Alectoris rufa*: reduced adult and chick survival, fertilization rate, and immune response), Nile tilapia (*Oreochromis niloticus*: extensive disintegration of testicular tissue and changes to gonads), Medaka (*Oryzias latipes*: juvenile stress led to ectoparasite infestation), and black-spotted pond frogs (*Rana nigromaculata*: DNA damage at very low concentrations)^20^. To our knowledge, no information is available on potential effects on large mammals, such as white-tailed deer (*Odocoileus virginianus*).

Over the past decade, morphological and developmental abnormalities have been documented in white-tailed deer in west-central Montana. Of 254 male deer of various ages, 67% showed genital developmental abnormalities such as mispositioned and undersized scrota and ectopic testes^21^; these abnormalities were documented for accident-killed and injured cervids^21^. Hoy *et al*.^21^ suggested that genital anomalies could be caused by endocrine disrupting pesticides but stated that, based on the information available, no cause and effect could be justified. In addition, from 2000 to 2009, brachygnathia superior (i.e., mandibular prognathia or underbite) increased from 0% to 70% for white-tailed deer that were collected from west-central (accident killed) and throughout (hunter harvested) Montana^22^. Underbite is a characteristic of congenital hypothyroidism, which has been documented in South Dakota^23^, and is nearly always associated with fetal thyroid hormone function^22^, but the cause has not been empirically determined for this observation.

We hypothesized that imidacloprid would have sub-lethal and potentially lethal effects on adult female and fawn white-tailed deer. We predicted that adult females, especially in the high treatment group, would have reduced Free Triiodothyronine (FT3) and Free Thyroxine (FT4) levels, presence of imidacloprid in milk, and reduced activity associated with exposure to imidacloprid. We also predicted that fawns exposed to imidacloprid at relatively high treatment levels would have abnormal genital organs, lowered FT3 and FT4 levels, reduced activity, and a high prevalence of under bite.

## Methods

Our research study was conducted at the South Dakota State University Wildlife and Fisheries Captive Research Facility in Brookings County, South Dakota (44°20′N, 96°47′W). This facility housed white-tailed deer (beginning in about 1998) on 4 ha; the facility is double fenced with 3-m high woven wire. The facility is situated adjacent to agricultural fields normally planted to corn or soybeans, and is surrounded by a shelterbelt of trees. Mean annual temperatures were 7.4 °C (ranged from −28.8 °C and 34.4 °C) and 7.8 °C (−35 °C to 32.8 °C) in 2015 and 2016, respectively. Additionally, daily annual precipitation was 0.18 cm (0.03 cm to 5.2 cm) and 0.2 cm (from 0.3 cm to 7 cm) in 2015 and 2016, respectively. Finally, daily annual snowfall was 0.3 cm (0.3–17.8 cm) and 0.3 cm (0.3–11.4 cm) in 2015 and 2016, respectively^24^.

Twenty adult white-tailed deer were randomly selected for the experiment and bred; parturition occurred in May and June of each experimental year. Adult females were separated into four treatments (care was taken to separate adult females so that age and weight were uniformly distributed): control (n = 4), low (n = 4), moderate (n = 5), and high (n = 7) (the moderate and high treatment groups had a larger sample size to reduce the standard error for our response variable). Deer were housed in pens of similar size (control = 130 m^2^/deer, low = 175 m^2^/deer, moderate = 123 m^2^/deer, high = 112 m^2^/deer in 2015 and 165 m^2^/deer in 2016). All deer were fed rations that included soy hulls, shelled corn, and alfalfa hay *ad libitum*.

Adult females were administered aqueous imidacloprid (Product # 37984, Sigma Aldrich St. Louis, MO) from May until October to mimic free water availability within the Dakotas. We added 0 ng/L, 1,500 ng/L, 3,000 ng/L, and 15,000 ng/L of imidacloprid to the control, low, moderate, and high treatments, respectively. The low and moderate concentrations were similar to wetland levels found in groundwater in Wisconsin (detected in 24% of the groundwater sample and ranged from 260–3,340 ng/L); however, they were greater than levels found in rural streams in Iowa (detected in 23% of streams sampled and ranged from <2–42.7 ng/L) or in Canadian (Saskatchewan) wetlands (detected in 12% of wetlands and ranged from 7.1–256 ng/L)^25^. Our high treatment was intended to invoke an effect and therefore, was much greater than documented in free water. Deer were provided with a 60.6 L tub that contained 37.8 L of water treated with the appropriate amount of imidacloprid depending on the group (control, low, moderate, high). Deer consumed the water treated with imidacloprid *ad libitum*. Water levels were checked daily and refilled with the appropriate imidacloprid treated water when empty or less than 3 cm from the bottom (every 1–2 d) of containers. When refilling occurred, each tub was rinsed thoroughly and excess water was poured into 189 L tubs provided by the SDSU Environmental Health and Safety office.

Fawns born to adult females in the study were included in our experiment. On the day of parturition, each fawn was handled minimally with gloves to determine body mass and sex; fawns also were fitted with ear tags. To mimic natural water availability, fawns were not prevented from consuming the imidacloprid in water. Facilities and techniques for research were approved by the South Dakota State University Institutional Animal Care and Use Committee (IACUC number 15–055 A**)** and followed guidelines by the American Society of Mammologists^26^.

### Solution consumed

During experiments, water tubs housing aqueous imidacloprid were weighed daily to determine the volume of water consumed per group. Analysis of variance (ANOVA) was performed to compare water consumption among treatment and control groups with date used as a covariate. To detect imidacloprid concentrations as the imidacloprid water was consumed, a 3-d experiment was conducted. On day 1, the appropriate treatment or control group concentrations were created in five, 63 L galvanized tubs. On day 2, 50% of the water was removed from all tubs. On the third day, nearly all water was removed from the tubs, leaving only enough water to coat the bottom of tubs. Samples (15 mL) were collected daily from each tub. This procedure mimicked water level reductions due to deer consumption. Imidacloprid samples were analyzed using ELISA (enzyme-linked immunosorbent assay; Abraxis, Warminister, PA; See Section 4.7 for procedures).

### Collection of blood samples

Blood samples were collected from adult females and fawns in treatments using BD Vacutainer Serum tubes (Becton, Dickinson, and Company, Franklin, NJ). We collected up to 12 mL of blood from the saphenous vein approximately monthly during treatments while deer were held in a chute (Priefert Wildlife Equipment Deer Chute; Priefert®, Mount Pleasant, TX). We collected blood samples (1–10cc from the saphenous or jugular) from fawns twice; 1 wk after parturition and at approximately 5 mo of age. Blood samples were refrigerated until processed to extract serum (1 h to 2 d). Upon reaching the lab, blood samples were centrifuged (Ultra-8V; LW Scientific, Lawrenceville, GA) for 15 min at 280 × g to separate serum for testing FT3 (free triiodothyronine) and FT4 (free thyroxine) hormones.

FT3 and FT4 thyroid hormones reflect the ability of the deer to utilize body fat reserves, regulate basal metabolic rate, and control thermal regulation^27^. Serum from blood samples was transferred to labeled 1.5 mL microcentrifuge tubes (BrandTech® Scientific Inc., Essex, CT), sealed, and frozen at −20 °C. These samples were then overnighted to the Diagnostic Center for Population and Animal Health at Michigan State University (Lansing, MI) for FT3 and FT4 testing. These assays were performed with commercially available solid-phase radioimmunoassay kits (FREE T3 Solid Phase Component System and Free T4 Solid Phase Component System, MP Biomedicals Diagnostics Division Orangeburg NY 10962). The volumes of sample, assay standards, and radioligand were used according to the manufacturer’s protocol. Incubation times for free T3 and free T4 assays were 2.5 h and 1.5 h, respectively, at 37 °C.

### Behavioral Observations

Focal sampling of behavioral observations were collected on treatment and control groups prior to death. Behaviors included eat, lay, lay/groom, lay/ruminate, stand/ruminate, run, stand, stand/groom, stand/nurse, and walk; for fawns, the behaviors lay/curl and lay/sleep also were recorded. Observations were conducted in 1 h blocks using an ethogram^28^. During time blocks, occurrences of behaviors were tallied and the duration of each behavior (in s) was recorded. Observations occurred between 6:00 and 16:00. In each session, an adult female or fawn was randomly chosen (without replacement) from each treatment and control group (n = 28 h for 2016 fawns and n = 21 h for adult females).

### Necropsies

All deer in the experiment (adult females and fawns) were euthanized and subsequently necropsied using IACUC approved protocols. Fawns were euthanized at the end of each field season (October 2015 and 2016) and adult females were euthanized at the completion of the study (October 2016). Adult females and fawns were first tranquilized using xylazine (Bayer, Englewood, Colorado) and telezol (Zoetis, Parsippany-Troy Hills, New Jersey) when held in a Priefert deer chute and, once immobilized, were euthanized using euthanasia solution (MWI Veterinary Supply, Boise, ID) according to manufacturer’s suggested dosage. Once does and fawns were euthanized, they were frozen at −20 °C. All fawns and does in the experiment were necropsied at the South Dakota Animal Disease Research and Diagnostic Laboratory, South Dakota State University, Brookings, South Dakota.

Necropsies were performed by Dr. David Knudsen (assisted by E. Hughes Berheim). Liver, brain, spleen, and genital organs were extracted, weighed, and 2.54 cm^3^ samples were collected. Additionally, we collected fawn jawbones to determine length. Organ samples were then frozen at −20 °C until they could be analyzed using ELISA.

### ELISA Testing

Imidacloprid levels were determined for each organ collected. Brain, liver, spleen, and genital samples were removed from the freezer, and a portion of each organ (0.5–0.75 g) was minced using a sterilized scalpel and placed into a polypropylene micro centrifuge tube. Water was added to the tube at a ratio of 1 mL:1 g tissue sample. Each mixture was shaken using a vortex (Thermo Scientific), heated in an 80 °C water bath for 10 min, and frozen at −20 °C. Frozen mixtures were thawed and centrifuged (Centrifuge 5424, Eppendorf) at 21,130 g for 1 min. The liquid was extracted and placed into separate micro centrifuge tubes; remaining solids in the organ samples and remaining liquid were refrozen. Liquid samples were vortexed and a 25 μL portion was extracted and placed into a separate microcentrifuge tube. The excess liquid also was stored frozen. The remaining liquid was mixed with 25 μL of water, vortexed for 5 s, and centrifuged for 2 s in preparation for the ELISA assay.

All samples were read at 450 nm using a microplate reader (uQuant, Biotek Instruments, Winooski, VT). Each plate had at least two standard curves of purified imidacloprid (Product number: 37894 SIGMA-ALDRICH, St. Louis, MO, USA). In preparation for the standard curve on each plate, samples from negative adult females were mixed together to account for the matrix effect of the organs and a stock solution of imidacloprid was created at 0.0, 0.03, 0.06, 0.13, 0.25, 0.5, 1.0, and 2.0 ppb. The standard curve on the ELISA plate contained 25 µL control organ in solution with 25 µL of the stock solution of imidacloprid, creating eight wells with concentrations that comprised one standard curve. We were unable to use the control organs in our standard curve because our experiment was unintentionally contaminated with imidacloprid; therefore, we used the deer sample with the lowest absorbance value as our baseline for quantifying imidacloprid quantities. We optimized the tissue preparation approach for this ELISA on solid samples using peer-reviewed methods^29–34^.

### Analysis

Data collected in experiments were analyzed using Systat 13 (Systat Software Inc., San Jose, CA). Male and female fawn organ concentrations for those fawns that survived versus those that died were compared using t-tests. Our ELISA results indicated that there was contamination of our control group. As a consequence, in addition to ANOVA, ordinary least square (OLS) linear regression was used to assess relationships between imidacloprid concentrations in all organ samples and the response variables birth weight, fawn age, FT3, FT4, jawbone length, and organ weights; alpha was set at 0.05.

Data collected on behavioral observations for adult females and fawns were analyzed separately but combined over observation period (morning and afternoon). Furthermore, we separated deer into three groups (high, moderate, and low) based on organ imidacloprid concentrations. Finally, we used Chi-square tests to determine significant differences among behaviors observed in high, moderate, and low imidacloprid groups for adults and fawns. If Chi-square tests were significant, we used confidence intervals (90%) to assess which behaviors differed among groups (high, moderate, and low).

## Results

### Doe and Fawn Survival

A total of 24 and 39 fawns was born in 2015 and 2016, respectively. In 2015, 12 of the fawns were born in August and September due to late breeding. Number of single, twin, triplet, and quadruplet litter sizes, respectively, per treatment were 1, 4, 1, 1 (control), 5, 3, 0, 0 (low), 2, 3, 2, 0 (moderate), and 2, 7, 2, 0 (high); 3 fawns were found outside treatment pens and were not included in analyses. Sex ratio (females:males) of fawns was 0.46:0.54 and did not differ by treatment (X^2^_3_ = 2.98, p = 0.394). In 2016, a control female died and was replaced with another adult female, totaling 21 adult females in our experiment. Fawn and adult female survival decreased over the two field seasons: survival of fawns was 75% and 62% in 2015 and 2016, respectively. Of 20 adult females in 2015, 0% died (100% survival); in 2016, 19% of 21 adult females died (n = 4, 81% survival). Survival of fawns did not differ (p > 0.05) between 2015 and 2016. Additionally, sample size for adult females was too small to evaluate change in survival between the two field seasons.

### Imidacloprid Solution Consumption

Water consumption rates in 2015 and 2016 were monitored, and daily consumption was recorded. There were significant interactions in water consumption between treatment and date in 2015 (F_15, 436_ = 2.22, p = 0.01) and 2016 (F_15, 555_ = 2.19, p = 0.01). In 2015, when the control was removed from the analysis, date was still significant (F_5, 327_ = 21.48, p = 0.01) relative to consumption; however, water consumption per adult female was similar across treatments (F_2, 327_ = 0.60, p = 0.55), indicating the control group consumed significantly more water than the treatment groups. In 2016, when excluding the control group, the high treatment group consumed less water per adult female than the low and moderate groups (F_2, 421_ = 12.83, p = 0.01), even though consumption of water increased throughout the field season (F_5, 421_ = 14.25, p = 0.01) (Table 1).

**Table 1:** Water consumed by deer in treatments in 2015 and 2016 field seasons (May to October).

Group	Date	Total Liters Consumed (SEM)	Average Liters Per Day (SEM)	Average Liters Per Doe (SEM)	Average Liters Per Fawn (SEM)
Low	2015	1616.7 (0.61)	12.7 (0.61)	3.3 (0.2)	1.72 (0.1)
Moderate	2015	2345.9 (0.78)	17.5 (0.78)	3.5 (0.2)	2.42 (0.1)
High	2015	2806.4 (1.13)	20.8 (1.13)	3.1 (0.2)	3.98 (0.2)
Control	2015	2574.1 (0.78)	19.5 (0.78)	5.2 (0.2)	4.23 (0.3)
Low	2016	2216.5 (9.7)	17 (0.85)	4.2 (0.2)	2.78 (0.1)
Moderate	2016	2323.2 (8.9)	17.7 (0.78)	3.6 (0.1)	3.89 (0.2)
High	2016	2430.3 (13.2)	19.1 (1.2)	3.3 (0.2)	6.06 (0.3)
Control	2016	2295.2 (8.6)	18.4 (0.77)	4.7 (0.2)	4.81 (0.2)

Average liters consumed, average liters consumed per day, and average liters consumed daily per doe were recorded for each treatment and control group. Average water consumed daily by fawns is also included in the table as it was used as a covariate in the ANOVA analysis of water consumption between treatment and control groups.

### Necropsy Data

Organ weights were collected from adult females and fawns, and jawbone measurements were collected solely from fawns. Adult females had mean organ weights of 159 ± 8 g for brain, 809 ± 104 g for liver, 388 ± 41 g for spleen, and 87 ± 28 g for genitals. Mean organ weights of fawns were 106 ± 3.9 g for brain, 413 ± 37 g for liver, 102 ± 11.4 g for spleen, and 6 ± 0.9 g for genitals. Female fawn mean organ weights were 96 ± 6 g for brain, 342 ± 55 g for liver, 95 ± 18 g for spleen, and 3 ± 0.6 g for genitals. Male fawn mean organ weights were 115 ± 5 g for brain, 479 ± 48 g for liver, 109 ± 14 g for spleen, and 9 ± 1 g for genitals (Table 2). Average jawbone length results were 13.8 ± 0.4 cm.

**Table 2:** Mean organ (brain, liver, spleen, and genital) weights (g) of adult females and fawns including standard error.

	Brain (g) (SEM)	Liver (g) (SEM)	Spleen (g) (SEM)	Genital (g) (SEM)
Adult female	161 (8)	1015 (63)	408 (40)	64 (15)
Fawn	105 (3.9)	432 (35)	103 (11)	6 (0.8)
Male fawn	115 (5)	479 (47)	109 (14)	9 (1)
Female fawn	95 (6)	385 (53)	99 (17)	3 (0.6)

Sample sizes are as follows: adult female n = 21, fawn n = 61 for the brain, spleen, and genital and n = 62 for the liver, male fawns n = 30 for brain, spleen, genital, and n = 31 for the liver, and female fawns n = 31 (all organs).

### ELISA Results

ELISA results indicated imidacloprid was found in the control group organs (Table 3), indicating that our treatments were contaminated; there were no significant differences across treatments for liver (F_3,18_ = 0.511, p = 0.14), brain (F_3,18_ = 0.058, p = 0.388), genital (F_3,18_ = 0.17, p = 0.286), or spleen (F_3,18_ = 0.17, p = 0.328) in adult females, or liver (F_3,34_ = 0.04, p = 0.943), brain (F_3,35_ = 0.018, p = 0.576), genital (F_3,34_ = 0.05, p = 0.707), or spleen (F_3,35_ = 0.20, p = 0.199) in fawns; gender of fawns was not significant (p > 0.07) in these analyses. However, imidacloprid concentration in spleen samples of adults approached significance (r = 0.36, p = 0.06) when regressed versus control and treatment categories. Nevertheless, this changed our focus from separating ELISA results by treatments to viewing the results relative to concentration of imidacloprid. Mean imidacloprid values in organs for all adult females were 0.42 ± 0.07 ng/g for liver, 0.06 ± 0.05 ng/g for brain, 0.11 ± 0.04 ng/g for spleen, and 0.69 ± 0.05 ng/g for genital (Table 3). Mean imidacloprid values in organs for female fawns were 0.42 ± 0.06 ng/g for liver, 0.03 ± 0.02 ng/g for brain, 0.21 ± 0.05 ng/g for spleen, and 0.26 ± 0.04 ng/g for genital (Table 3). Mean imidacloprid values in organs for male fawns were 0.55 ± 0.07 ng/g for liver, 0.05 ± 0.02 ng/g for brain, 0.19 ± 0.04 ng/g for spleen, and 0.15 ± 0.03 ng/g for genital (Table 3).

**Table 3:** Average imidacloprid levels in organs (ng of imidacloprid per gram of tissue) liver, brain, spleen, and genital in adult females (AF, n = 21), fawns (n = 65), female fawns (FF, n = 32), and male fawns (MF, n = 32) per treatment and control groups.

Age/Sex	Group	Survived/died	Liver (ng/g) (SEM)	Brain (ng/g) (SEM)	Spleen (ng/g) (SEM)	Genital (ng/g) (SEM)
AF	Control	All	0.351 (0.09)	0.222 (0.22)	0.012 (0.01)	0.388 (0.12)
AF	Low	All	0.133 (0.04)	0	0.077 (0.05)	0.380 (0.11)
AF	Moderate	All	0.495 (0.18)	0.010 (0.01)	0.111 (0.11)	0.287 (0.08)
AF	High	All	0.590 (0.12)	0	0.188 (0.10)	0.210 (0.06)
AF	All	Died	0.153 (0.04)	0.277 (0.21)	0.030 (0.02)	0.191 (0.13)
AF	All	Survived	0.487 (0.08)	0.003 (0)	0.124 (0.05)	0.330 (0.04)
AF	All	All	0.423 (0.07)	0.055 (0.05)	0.106 (0.04)	0.694 (0.05)
FF	Control	All	0.416 (0.06)	0.058 (0.03)	0.156 (0.04)	0.273 (0.04)
FF	Low	All	0.430 (0.05)	0.053 (0.02)	0.114 (0.02)	0.402 (0.04)
FF	Moderate	All	0.357 (0.05)	0	0.126 (0.02)	0.174 (0.02)
FF	High	All	0.426 (0.12)	0.008 (0)	0.294 (0.13)	0.222 (0.04)
FF	All	Died	0.443 (0.09)	0	0.268 (0.06)	0.219 (0.03)
FF	All	Survived	0.401 (0.07)	0.044 (0.03)	0.177 (0.08)	0.290 (0.06)
FF	All	All	0.417 (0.06)	0.028 (0.02)	0.210 (0.05)	0.264 (0.04)
MF	Control	All	0.681 (0.10)	0.065 (0.02)	0.223 (0.03)	0.102 (0.03)
MF	Low	All	0.350 (0.04)	0	0.037 (0.01)	0.168 (0.05)
MF	Moderate	All	0.566 (0.08)	0.044 (0.02)	0.252 (0.07)	0.148 (0.04)
MF	High	All	0.532 (0.09)	0.057 (0.04)	0.176 (0.06)	0.157 (0.03)
MF	All	Died	0.654 (0.08)	0.006 (0)	0.489 (0.07)	0.259 (0.04)
MF	All	Survived	0.518 (0.08)	0.057 (0.03)	0.116 (0.03)	0.115 (0.03)
MF	All	All	0.553 (0.07)	0.046 (0.02)	0.193 (0.04)	0.146 (0.03)
Fawn	All	Died	0.528 (0.04)	0.002 (0)	0.342 (0.03)	0.232 (0.02)
Fawn	All	Survived	0.463 (0.05)	0.051 (0.02)	0.144 (0.04)	0.200 (0.03)

AF, FF, and MF are also separated into averages for those that were dead, and alive at the end of the experiment, and the sum of all AF, FF, or MF in our study.

### Analyses

**Figure 1:**
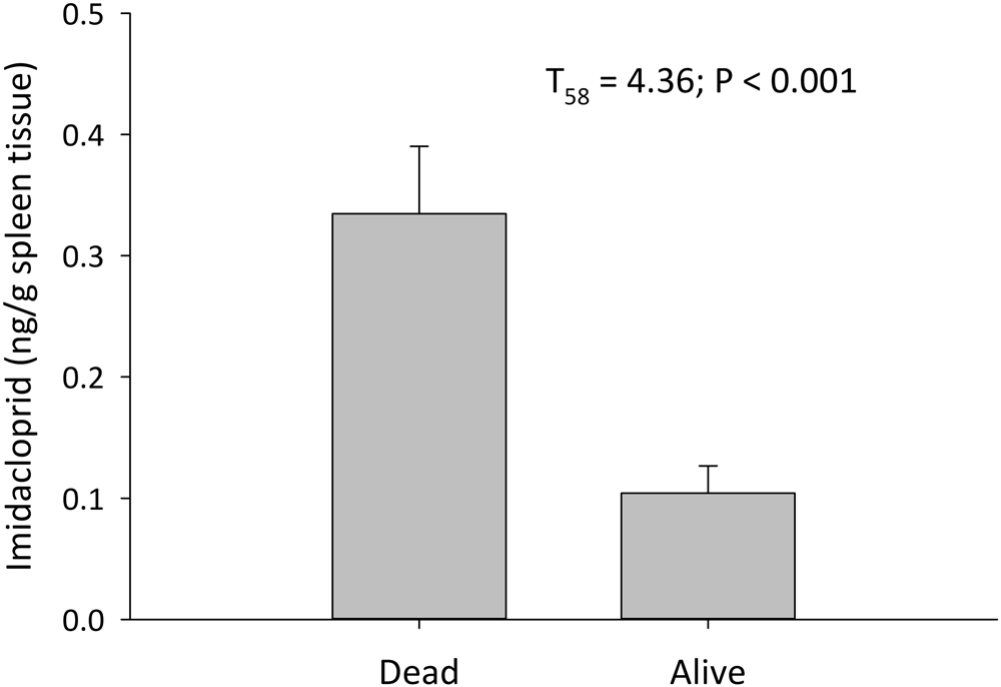
Average Imidacloprid levels (ng/g) in spleen tissue of 2015 and 2016 fawns (n = 62) that died prematurely compared to those that survived. Imidacloprid levels differed between those that were dead compared to alive.

Spleen concentrations of imidacloprid were significantly higher (T_59_ = 2.76, p = 0.007) in fawns that died compared to the fawns that survived. However, an outlier of 1.49 ng/g of spleen tissue was removed from analyses (mean of data with outlier 0.20, range 0–1.49; mean of data without outlier 0.18, range 0–0.91 ng/g of tissue); the revised result also was significant (T_58_ = 4.36, p < 0.001) (Fig. 1). The fawn with this high spleen imidacloprid concentration survived, which was not consistent with the overall trend in the data. Mean imidacloprid in spleens of fawns that died was 0.33 ± 0.26 ng/g whereas imidacloprid in spleens of fawns that survived averaged 0.10 ± 0.14 ng/g. Birth weight was not correlated with imidacloprid levels in any of the organs evaluated (Table 4). Fawn body weight at death was negatively correlated with imidacloprid levels in the spleen (F_1,55_ = 8.22, p = 0.005) and genital organs (F_1,56_ = 4.26, p = 0.04) (Table 4). Fawn age at death was correlated with imidacloprid levels in the spleen (F_1,57_ = 10.5, p = 0.002) but not in any of the other organs evaluated (Table 4). Adult female FT3 and FT4 values were not correlated with imidacloprid levels in organs (Table 4). Fawn FT3 values were not correlated with imidacloprid concentrations in organs; however, FT4 values in fawns were negatively correlated (F_1,39_ = 7.48, p = 0.0092, Table 4) with spleen imidacloprid concentrations. Adult female organ weights were negatively correlated with imidacloprid concentrations in genitals (F_1,19_ = 5.00, p = 0.04) but not with other organ levels evaluated. Fawn organ weights were negatively correlated with spleen (F_1,57_ = 8.78, p = 0.0044) and genital (F_2,54_ = 5.55, p = 0.021) (Table 4) imidacloprid concentrations. Fawn jawbone length was negatively correlated with imidacloprid values in the spleen (F_1,57_ = 9.98, p = 0.002) but not with other organ concentrations (Table 4).

**Table 4:** Results of regression analyses for imidacloprid concentrations in organ samples and physical results: birth weight, fawn body weight, fawn age, FT3 and FT4, organ weights, fawn jawbone length.

Physical Responses	Brain	Liver	Spleen	Genital
**Imidacloprid Concentration**				
Birth Weight	F_1,60_ = 0.04, P = 0.83	F_1,60_ = 0.25, P = 0.61	F_1,58_ = 1.25, P = 0.26	F_1,59_ = 0.08, P = 0.77*
Fawn Body Weight at Death	F_1,57_ = 0.98, P = 0.32	F_1,56_ = 0.35, P = 0.55	F_1,55_ = 8.22, P = 0.0058*	F_1,56_ = 4.26, P = 0.04
Fawn Age (in Days)	F_1,59_ = 1.78, P = 0.18	F_1,60_ = 0.0008, P = 0.97	F_1,57_ = 10.5, P = 0.0019*	F_1,58_ = 1.71, P = 0.19
AF FT3	F_1,19_ = 2.96, P = 0.10	F_1,19_ = 0.04, P = 0.85	F_1,19_ = 1.89, P = 0.18	F_1,19_ = 0.04, P = 0.83
AF FT4	F_1,19_ = 4.1, P = 0.06	F_1,19_ = 0.09, P = 0.76	F_1,19_ = 1.30, P = 0.27	F_1,19_ = 0.57, P = 0.46
Fawn FT3	F_1,39_ = 0.41, P = 0.52	F_1,39_ = 2.9, P = 0.09	F_1,38_ = 0.74, P = 0.39	F_1,39_ = 0.20, P = 0.65
Fawn FT4	F_1,40_ = 0.01, P = 0.90	F_1,40_ = 0.0002, P = 0.98	F_1,39_ = 7.48, P = 0.009*	F_1,40_ = 0.017, P = 0.89
AF Organ Weights	F_1,19_ = 0.04, P = 0.84	F_1,19_ = 1.15, P = 0.3	F_1,19_ = 0.29, P = 0.6	F_1,19_ = 5.0, P = 0.04*
Fawn Organ Weights	F_1,59_ = 2.42, P = 0.12	F_1,60_ = 0.10, P = 0.75	F_1,57_ = 8.78, P = 0.004*	F_1,58_ = 5.55, P = 0.02*
Fawn Jawbone Length	F_1,59_ = 1.5, P = 0.22	F_1,60_ = 0.11, P = 0.73	F_1,57_ = 9.98, P = 0.002*	F_1,58_ = 2.38, P = 0.12

P-values were considered significant when < 0.05.

^*^Indicates P-values that are significant and indicates a negative correlation so as imidacloprid increases the physical response decreases.

Imidacloprid concentrations in spleen were correlated with fawn survival; therefore, we explored relationships between spleen imidacloprid concentrations and deer behavior. Adult female (n = 21) imidacloprid concentrations in spleen were separated into low (n = 12, range = 0), moderate (n = 4, range 0.056–0.224), and high groups (n = 5, range = 0.248–0.909); the duration of behaviors were compared among groups (all spleens that had 0 ppb concentration were placed in the low group). The low imidacloprid group differed (90% CI) from the high group in the behaviors eat (groups; high = 2.4%, low = 6%), lay (high = 27%, low = 19%), lay/groom (high = 7%, low = 3%), stand/ruminate (high = 1%, low = 2%), run (high = 1%, low = 5%), and stand/groom (high = 8%, low = 5%) indicating that adult deer in the low group had higher activity levels than those in the high group. The moderate group also differed from the low group in the behaviors eat (group; moderate = 10%, low = 6%), lay (moderate = 4%, low = 19%), lay/ruminate (moderate = 2%, low = 5%), stand/ruminate (moderate = 1%, low = 2%), run (moderate = 1%, low = 5%), stand (moderate = 34%, low = 23%), stand/groom (moderate = 13%, low = 5%), and stand/nurse (moderate = 0%, low = 2%); indicating variation in behavior between the two groups (Table 5).

**Table 5:** Behavioral observations closest to individual adult female (AF: n = 21) and fawn (n = 38) deaths (time ranged from 1 week to 2 months) were compared to their spleen imidacloprid concentrations.

A.F./Fawn	Group	Eat	Lay	Lay/Curl	Lay/Slp	Lay/Grm	Lay/Rum	Sta/Rum	Run	Stand	Sta/Grm	Sta/Nur	Walk
AF	High	2%*	27%*	N/A	N/A	7%*	4%	1%*	1%*	25%	8%*	1%	24%
AF	Moderate	10%*	4%*	N/A	N/A	3%	2%*	1%*	1%*	34%*	13%*	0%*	32%
AF	Low	6%	19%	N/A	N/A	3%	5%	2%	5%	23%	5%	2%	30%
Fawn	High	7%	43%^	8%	2%	7%	7%	N/A	0%^	16%^	2%^	N/A	9%^
Fawn	Moderate	12%^	20%	1%^	1%	15%^	5%	N/A	3%	21%	5%	N/A	17%
Fawn	Low	8%	24%	6%	2%	8%	5%	N/A	4%	22%	6%	N/A	15%

Not all fawns had observations collected as: 1) fawn observations were only collected in 2016 and 2) some fawns died prior to an observation being completed. Behavioral observations are separated into three groups (low, moderate, high) according to spleen organ concentrations (with the high group having the greatest imidacloprid levels and the low group having the lowest). Behavioral observations eat, lay, lay/groom (lay/grm), lay/ruminate (lay/rum), stand/ruminate (sta/rum), run, stand, stand/groom (sta/grm), stand/nurse (sta/nur), and walk (additionally for fawns the behaviors Lay/Curl and Lay/Slp (lay/sleep)) percentages were compared between spleen organ imidacloprid concentrations.

*Percentages that are significantly (90%CI) different than the low group percentages in the spleen for adult females.

^Percentages that are significantly (90%CI) different than the low group percentages in the spleen for fawns.

Fawn spleen concentrations of imidacloprid (n = 38) also were placed in low (n = 20, range = 0), moderate (n = 9, range = 0.053–0.121), and high (n = 9, range = 0.148–0.786) groups and the durations of particular behaviors were compared among groups. The low group differed (90% CI) from the high group in the behaviors lay (group; high = 43%, low = 24%), run (high = 0%, low = 4%), stand (high = 16%, low = 22%), stand/groom (high = 2%, low = 6%), and walk (high = 9%, low = 15%); indicating that the high group was less active than the low group. The moderate group also differed from the low group in the behaviors eat (group; moderate = 12%, low = 8%), lay/curl (moderate = 1%, low = 6%), and lay/groom (moderate = 15%, low = 8%); indicating variation in behavior between the two groups (Table 5).

## Discussion

Our study provides the first overview of effects of imidacloprid on white-tailed deer. We documented that deer in our experiment avoided imidacloprid-contaminated water. Moreover, we discovered that fawns that died during our experiment had greater concentrations of imidacloprid in spleens compared to those that survived. Fawns with relatively high concentrations of imidacloprid in spleen and genital organs also tended to be smaller and less healthy than those with relatively low concentrations of imidacloprid in these organs. Finally, our study provides support for reduced activity of adult and fawn white-tailed deer with relatively high concentrations of imidacloprid in spleens.

ELISA results indicated that our control experimental tissues were unintentionally contaminated with imidacloprid. Potential sources of contamination included seed-treated food and vegetation. Deer were fed soy hulls and a corn, oats, and distiller’s mixture *ad libitum*. Unfortunately, it was unknown if the soybeans and grains fed to our deer were from imidacloprid-treated plants. However, corn and soybeans are commonly (≥94% of U.S. corn, ~50% of U.S. soybeans^12^) coated with one of the neonicotinoid active ingredients: clothianidin, imidacloprid, or thiamethoxam^13^. Additionally, deer in our study would often reach through the fence to browse on natural vegetation. The fields adjacent to the captive wildlife facility were a matrix of agricultural crops with a corn field about 50 m north of the facility. It is unknown what pesticides were used on the corn, but it is likely that there was a seed treatment of imidacloprid or clothianidin. In Indiana, neonicotinoid dust was documented to disperse as far as 100 m from the site^35^. Imidacloprid from fields could be washed off during rain events and be absorbed by other plants, although this transfer is poorly understood^19,36^. Therefore, uptake of imidacloprid by vegetation adjacent to the facility is a likely source of this contamination.

Water containing imidacloprid was avoided by deer in treatments in our experiment as evidenced by variable concentrations of the neonicotinoid in captive deer. Deer that avoided consumption of treated water likely drank rain water, which was available (up to 0.3 m deep) after storm events during our experiment. Research on cervid avoidance of imidacloprid is unavailable, but avoidance of imidacloprid has been recorded in red-legged partridge (*Alectoris rufa*) when offered treated seeds^37^. Other animals detect and avoid toxins in their diets; for example, kudus (*Tragelpahus imberbis*), impalas (*Aepyceros melampus*), and goats (*Capra aegagrus hircus*) in South Africa avoided plants with 5% condensed tannins during the wet season^38^, likely due to the astringency of these compounds.

Significantly higher concentrations of spleen imidacloprid levels were found in fawns that died compared to those that survived. The spleen produces white blood cells that fight infection and synthesize antibodies^39^. Imidacloprid can reduce the production of spleen lymphocytes^40–42^, which results in an impaired immune system^20^. Therefore, immune suppression in our fawns caused by imidacloprid likely was a factor in their deaths. Complimentary results were found in the FT4 values that are a pre-cursor to FT3 hormone, which is instrumental in regulating basal metabolic rate and thermal regulation in deer^27^. FT4 was inversely correlated with imidacloprid in spleens of fawns. Reduced metabolic rate in fawns with relatively high concentrations of imidacloprid likely explain the lower activity documented in captive deer.

Imidacloprid values in brain were low to undocumented, which was surprising considering that the pesticide affects the central nervous system; we hypothesize that this could be due to an inability of the chemical to cross the blood-brain barrier. The California Environmental Protection Agency found that imidacloprid penetrates the blood-brain barrier. However, Gupta *et al*.^43^ found high imidacloprid quantities in rat liver, kidney, lung, and skin, but concentrations in the brain were low. Additionally, Krieger^44^ noted that the blood-brain barrier in vertebrates blocks access of imidacloprid to the central nervous system, which reduces toxicity.

Fawns had similar birth weights regardless of the level of imidacloprid in their organs. Similarly, in Sprague-Dawley rats, there were no differences in litter size or weight gain in the offspring whether or not mothers were given an intraperitoneal injection of imidacloprid^45^. Additionally, Gawade^41^ found no significant difference in weights of imidacloprid exposed Wister rat pups. However, imidacloprid levels in spleen and genital tissues were negatively associated with body weight in fawns at the time of death.

FT3 and FT4 results are indicative of basal metabolic rate and thermoregulation^27^. Fawn and adult female FT3 values were similar to those reported in other studies, but FT4 results were elevated compared to previous studies^27,46–48^. We do not believe that this was the result of imidacloprid, as this pesticide decreases thyroid function in rats^49^, Indian wild birds^50^, and fish^51^. Rather, the elevated FT4 values may be due to a combination of pregnancy in adult females, the time of year, and artificial feed. Hamr *et al*.^52^ found that thyroid hormones of artificially fed deer were elevated compared to deer that consumed natural browse. Additionally, this study also found that hormones were increased in the spring and summer. Bahnak *et al*.^53^ documented that pregnant, penned deer have elevated levels of thyroid hormones.

As imidacloprid increased in the spleen, we noted that FT4 levels and spleen size decreased. As stated previously, imidacloprid has been shown to decrease FT4 levels in other vertebrates^49–51^. Additionally, research on rats has shown that organ weights (specifically liver and spleen) decrease as imidacloprid treatment increases^54–56^. From our observations and previous research, we predict that imidacloprid is suppressing the immune function and size of the spleen.

As imidacloprid increased in the genital organs of fawns and adult females, weights of the genital organs decreased. Vohra and Khera^56^ found that, as oral consumption of imidacloprid increased, the ovaries of lab rats became smaller but the uterus increased in size. Additional research has shown that liver and spleen sizes will decrease as imidacloprid concentration increases; however, there was not an indication that the genital weight decreased^54–56^. Consequently, more research is needed to better understand how imidacloprid and other neonicotinoids affect reproductive tissues in mammals.

Behavioral observations indicated that high concentration of imidacloprid in the spleen resulted in less activity in adult females and fawns. This finding was similar to results on female rats and their offspring that showed significant decreases in grip time as imidacloprid concentrations from intraperitoneal injection increased, an indication of fatigue^45^. Rat movement was similarly impaired as imidacloprid (via oral consumption) increased^55,57^.

Samples of liver and spleen organs were collected from white-tailed deer brought to the NDGF Wildlife Health Laboratory for a variety of reasons (e.g., illegal harvest investigations, disease, deer-vehicle collisions) from 2009–2017 throughout North Dakota.; imidacloprid concentrations were evaluated in 367 samples using the same ELISA methods as in our captive experiment. Results indicated that levels of imidacloprid in liver samples were 2.8 times higher in free-ranging deer in North Dakota [average 1.32 (0.10)] than in livers of our captive deer [average 0.46 (0.03)], Table 6. Furthermore, concentrations of imidacloprid in spleen samples from free-ranging deer in North Dakota [0.60 (0.06)] were 3.5 times higher than those in spleens of captive deer [0.17 (0.02), Table 6] in our experiment. Deer exposure to imidacloprid averaged 52.3 ± 4.6% over the years 2009 to 2017. For those free-ranging deer in North Dakota exposed to imidacloprid, average concentrations in spleens increased (r = 0.22, p = 0.002) an average of 0.11 ng/g per year from 2009 to 2017. Furthermore, 77.5% of these deer had imidacloprid levels in spleens equal to or above 0.33 ng/g (i.e., mean level of imidacloprid in spleens of fawns in captivity that died in our experiment). These results indicate that wild populations of deer exposed to imidacloprid are potentially experiencing effects similar to those seen in our captive facility experiment; i.e., reduced activity in adult females and fawns, and specifically in fawns, decreased survival, size, and health. Consequently, additional research is needed to confirm these relationships in free-ranging deer in agricultural landscapes where imidacloprid and other neonicotinoid insecticides are utilized.

**Table 6:** Comparison of liver and spleen imidacloprid concentrations (ng/g of tissue) between North Dakota free-ranging deer (n = 367) and our captive facility deer (n = 86).

	Liver		Spleen	
	North Dakota	Captive	North Dakota	Captive
Maximum	8.42	1.36	6.61	1.48
Minimum	0	0	0	0
Average	1.32	0.46	0.60	0.18
STD	1.68	0.34	1.12	0.26
SEM	0.10	0.03	0.06	0.03

## Data Availability

Data are available for upload upon the publication of our manuscript.
